# Effects of LED light treatments on the bioactive composition and functional properties of pea (*Pisum sativum* L.) microgreens: a comparative analysis with sprouts

**DOI:** 10.3389/fpls.2026.1834435

**Published:** 2026-05-28

**Authors:** Suzana Pavlović, Betül Aydın, Erdi Can Aytar, Abidin Gümrükçüoğlu, Nikolija Krstić, Nevena Vidović, Marija Knez

**Affiliations:** 1 Group for Nutrition and Metabolism, Institute for Medical Research, National Institute of Republic of Serbia, University of Belgrade, Belgrade, Serbia; 2 Faculty of Science, Department of Biology, Gazi University, Ankara, Türkiye; 3 Faculty of Agriculture, Department of Horticulture, Uşak University, Uşak, Türkiye; 4 Medicinal-Aromatic Plants Application and Research Center, Artvin Coruh University, Seyitler Yerleşkesi, Artvin, Türkiye; 5 Center of Research Excellence in Nutrition and Metabolism, Institute for Medical Research, National Institute of Republic of Serbia, University of Belgrade, Belgrade, Serbia

**Keywords:** LED light treatment, microgreens, phytochemical profile, pisum sativum, sprouts

## Abstract

**Introduction:**

The concept of functional foods enriched with bioactive compounds, including polyphenols, flavonoids, and antioxidants, encompassing a diverse array of dietary items that extend beyond basic nutrition to confer specific health benefits, has received increasing attention in contemporary nutrition science. The study evaluated the effects of different LED light treatments on the morphological traits, pigment composition, antioxidant potential, and polyphenolic profile of pea microgreens and sprouts.

**Methods:**

Pea sprouts and microgreens were grown under different LED light spectra (blue, red, blue–red, cool white and darkness) in controlled growth chamber conditions, followed by evaluation of morphological parameters, photosynthetic pigments, antioxidant activity, and phenolic composition. Photosynthetic pigments were determined spectrophotometrically, total phenolics and flavonoids were analyzed using Folin–Ciocalteu and AlCl₃ assays, antioxidant capacity was evaluated by the DPPH method and phenolic profiling was performed using HPLC-DAD analysis.

**Results and conclusion:**

Results indicated substantial differences in fresh weight (FW) and dry weight content (%DW) under various light conditions. The highest growth was observed under blue-red (BR) light in sprouts and blue (B) light in microgreens (0.169 and 0.291 g FW, respectively). In sprouts, these responses are likely associated with light-induced photomorphogenic processes rather than fully developed photosynthetic activity, while microgreens, which possess functional chloroplasts, respond directly through photosynthesis. Cool white (CW) light resulted in the highest %DW in both growth stages (10.197% in sprouts and 11.651% in microgreens). Pigment content analysis showed that total chlorophyll concentrations were highest under BR light in both sprouts and microgreens (0.912 and 9.257 mg/g DW), with microgreens exhibiting elevated pigment levels overall. The total phenolic content (TPC) and total flavonoid content (TFC) varied across treatments, with the highest TPC value under blue light in sprouts (5.869 mg GAE/g DW) and CW light in microgreens (7.531 mg GAE/g DW). TFC was highest under BR light for both developmental stages (1.046 and 1.759 mg QE/g DW). Antioxidant capacity, measured by DPPH radical scavenging activity, indicated that CW and blue light promoted the strongest antioxidant potential in both sprouts and microgreens. Analysis of phenolic compounds revealed substantial variability, with gallic acid and epicatechin being dominant under different light treatments. Exploratory molecular docking analysis indicated that rosmarinic acid has the highest predicted binding affinity for xanthine oxidase among the tested compounds, suggesting that experimental validation of these hypothesis-generating findings is needed. Overall, the results indicate that specific LED light treatments significantly influence the growth characteristics and biochemical properties of pea sprouts and microgreens.

## 1 Introduction

The concept of functional foods, encompassing a diverse array of dietary items that extend beyond basic nutrition to confer specific health benefits, has received increasing attention in contemporary nutrition science. These foods are enriched with bioactive compounds, including polyphenols, flavonoids, and antioxidants. Bioactive compounds have the ability to modulate physiological processes, mitigate oxidative stress, and potentially reduce the risk of chronic ailments, including cardiovascular diseases, diabetes, and certain types of cancers (Jurek, 2022; Yuan et al., 2024). Legumes represent a diverse array of plant-based foods, with the pea (*Pisum sativum* L.) being particularly notable for its high nutritional density, encompasing high-quality plant-based proteins, dietary fibres, vitamins such as C and K, minerals like iron and potassium, and secondary metabolites such as phenolic acids and carotenoids, which collectively contribute to its anti-inflammatory and antioxidant properties (Mohamed and Loumerem, 2024; Sun et al., 2024). Concurrently, microgreens – defined as tender, immature greens harvested shortly after the emergence of the first true leaves – represent an innovative category within the domain of functional foods. They offer superior nutrient density compared to their mature counterparts, often exhibiting 4- to 40-fold higher concentrations of vitamins, minerals, and phytochemicals per unit weight (Harsha et al., 2025). The significance of these foods lies in their role as nutrient-rich additions to diets, thereby enhancing the bioavailability of health-promoting compounds while supporting sustainable eating practices. From an agricultural perspective, microgreens facilitate resource-efficient production using controlled-environment systems, short cultivation cycles (typically 7–21 days), minimal land use, and adaptability to vertical farming, thereby contributing to urban food security and climate-resilient agriculture (Taşcı Durgut et al., 2025).

Plants use light as the main source for photosynthesis, which regulates numerous processes related to plant growth, morphology, and secondary metabolism (Manivannan et al., 2015; Lobiuc et al., 2017; Ali et al., 2019; Paradiso and Proietti, 2022). Through specific photoreceptors, plants respond to both the intensity and the spectral composition of light. In recent years, LED (light-emitting diode) lights, which are more energy-efficient and allow precise control of spectral quality, intensity, and photoperiod, have been increasingly used to grow plants under controlled conditions in growth chambers (Bantis, 2021). The influence of different light spectra depends on the plant species, so it must be optimized for each species and specific working conditions (Liang et al., 2021). LED spectral manipulation affects not only shoot elongation but also the biosynthesis of secondary metabolites in pea microgreens (Wu et al., 2023a). Numerous studies have demonstrated that blue or mixed red-blue LED light significantly increases the accumulation of pigments, flavonoids, and phenolics, while red light promotes growth and early biomass accumulation (Gao et al., 2022; Zhao et al., 2024). This enhancement is linked to the upregulation of light-responsive phenylpropanoid pathway enzymes. The combination of red and blue light often results in synergistic effects, improving both growth and photosynthetic efficiency in pea microgreens. Moreover, supplementing white LEDs can provide a broader spectrum, which further supports balanced growth and metabolite accumulation (Wojciechowska et al., 2015). This makes spectral optimization an attractive strategy for improving the nutritional quality of pea microgreens, which require an optimal combination of light spectrum, intensity, and photoperiod length for optimal growth and quality. High intensity increases biomass production, but excessive intensity does not necessarily increase the accumulation of bioactive compounds (Balázs et al., 2023). The optimal combination for pea microgreens is less well characterized than that for other species, as most research focuses on sprouts or seedlings. Standardization of experimental reporting (e.g., spectrum, intensity, photoperiod) is inconsistent, limiting the reproducibility and comparability of results across studies (Balázs et al., 2023). Genetic diversity in pea significantly influences pigment and phenolic composition, and genotype × light interactions remain poorly documented (Wu et al., 2023b; Kyriacou et al., 2019a). In this context, pea microgreens represent an excellent model crop to investigate how different LED light spectra modulate both growth performance and biochemical composition.

Sprouts and microgreens represent distinct developmental stages, with heterotrophic sprouts relying primarily on seed reserves and microgreens exhibiting active photosynthesis due to functional chloroplasts. In grown sprouts, light is perceived through photoreceptors, triggering photomorphogenesis – a light driven developmental process independent of photosynthesis. This study aims to analyze both stages to assess how LED light treatments influence the transition from light signaling driven responses to fully photosynthetic growth and to investigate the effects of different LED light treatments on the growth and content of pigments, phenolics and flavonoids in pea microgreens, providing insights for designing optimized production systems that support both nutritional and functional food goals.

## 2 Materials and methods

### 2.1 Plant material and cultivation

The seeds of the commercial pea variety Kelvedon (Seme Semena, Belgrade, Serbia) were germinated on biodegradable jute fiber mats (China). After germination, the plants were grown under different LED light spectra (cool white-CW, red-R, blue-B, and blue: red combination 1:1-BR) and in the darkness (D). Each LED set was independently controlles using dimer (for light intensity) and a timer (for photoperiod regulation). The blue LED showed a narrow peak at 459 nm, the red LED peaked at 632 nm, and the cool white LED displayed a broad spectrum with a blue peak at 446 nm and wide emission from 480 to 650 nm, centred around 532 nm. The cool white LED emitted approximately 45–50% of photons in the blue region (400–500 nm), 35–40% in the green region (500–600 nm), and 10–15% in the red region (600–700 nm), whereas the monochromatic LEDs exhibited >90% of their emission within their respective spectral bands (blue LED in 400–500 nm, red LED in 600–700 nm). The blue:red (1:1) treatment was defined by photon flux density (PPFD), with equal contributions of blue and red photons. The effect of different light qualities on growth, photosynthetic pigment content, and antioxidant levels in pea microgreens was investigated. Darkness (D) was included as a developmental reference to represent growth without light-driven processes, enabling comparison between heterotrophic, seed reserve-driven growth (particularly in sprouts) and photoautotrophic responses, rather than as a physiological control. All plants were grown in a growth chamber at a temperature of 23 ± 2 °C under a long-day light regime (16 hours day, 8 hours night). Photosynthetic photon flux density (PPFD) measured at the top of the plants was 145 μmol/m²s¹ and the daily light integral (DLI) was 8.35 mol/m^2^d^1^. Fresh and dry weights (FW and DW) of 10 sprouts and microgreens were measured after 7 days of growth for sprouts and 14 days for microgreens. Length of microgreens was measured after 14 days of growth. DW was measured after lyophilization and expressed as % of FW (%DW). Absolute dry mass (ADM) was calculated as FW × %DW and expressed in g per sample.

### 2.2 Determination of photosynthetic pigments

The contents of photosynthetic pigments were measured after 7 days of growth for sprouts and 14 days for microgreens. Isolation and determination of chlorophyll and carotenoid content was carried out according to the method of (Brouers and Michel-Wolwertz, 1983). Chlorophyll (Chl A and Chl B) and carotenoid content (TCC) were determined spectrophotometrically (JENWAY 6850, Cole Palmer, IL, USA). Absorbance (A) was measured at three wavelengths: 470 nm (max absorption for carotenoids), 645 nm (max absorption for chlorophyll b), and 663 nm (max absorption for chlorophyll a). The total content of chlorophyll and carotenoids was calculated according to the formulas of (Lichtenthaler, 1987) and expressed in mg/g of dry sample weight.

### 2.3 Sample preparation for the determination of total flavonoids, phenols, and antioxidant potential

After 7 days of growth for sprouts and 14 days for microgreens, cleaned sprouts and microgreens were frozen at -20 °C for 24 h and then were lyophilised (Lyovac GT2, SRK-Systemtechnik GmbH, Riedstadt, Germany) until completely dried (approx. 48 h), ground to a fine powder, and stored at +4 °C until extraction. An ultrasound-assisted extraction method was used to extract phytochemicals from plant samples. Approximately 1 g of the sample was mixed with 20 mL of 80% methanol (v/v) and subjected to ultrasound treatment for 35 min at a maximum temperature of 30 °C and a frequency of 40 kHz. After that, the mixture was centrifuged for 5 minutes at 6000 rpm, and the supernatants were collected, filtered, and stored at +4 °C until analysis. The extracts were used to determine the total content of flavonoids, phenols, and antioxidant potential, as well as to perform HPLC-DAD analysis. Measurements were performed in triplicate.

### 2.4 Antioxidant activity assays

The total phenolic content (TPC) of the extracts was determined using the Folin–Ciocalteu method, a widely used spectrophotometric assay that facilitates the measurement of phenolic compounds. In this process, 200 μL of Folin-Ciocalteu reagent was added to the extract to ensure a homogeneous mixture was achieved. The mixture was then incubated at room temperature for 3 minutes. Following this, 1 mL of a 2% sodium carbonate solution (w/v) was added to terminate the reaction. The reaction was then incubated in the dark for an additional hour. Following the incubation period, the absorbance of the samples was measured at a wavelength of 760 nm, using a UV-visible spectrophotometer (Biotek, Epoch, USA). The TPC of the extracts was then determined using a calibration curve of gallic acid and expressed as gallic acid equivalents (mg GAE/g DW) (Singleton and Rossi, 1965).

The total flavonoid content (TFC) of the extract was determined by the AlCl_3_ method. A quantity of 0.05 mL of the extract was combined with an equal volume of a 2% aluminium chloride solution in methanol (w/v). After thoroughly mixing the reaction mixture, the mixture was allowed to elapse at room temperature for 10 minutes. The absorbances of the resulting yellow solutions were measured at a wavelength of 367 nm. The determination of TFC was accomplished through the utilisation of quercetin equivalents (mg QE/g DW) values (Osuna-Ruiz et al., 2016).

The capacity of the extract to scavenge free radicals was evaluated through the 1, 1-diphenyl-2-picrylhydrazyl (DPPH) assay, with certain modifications to the established procedure (Braca et al., 2001). The extract solutions were subjected to serial dilution using 80% methanol(v/v). A solution comprising 50 μL of the diluted sample was mixed with an equivalent volume of the DPPH solution. The control substance comprised 80% methanolic solution. The mixtures were then incubated in darkness at ambient temperature for 30 minutes. Following this, the absorbance value was measured at a wavelength of 517 nm. The following equation was used to determine the percentage of DPPH radical scavenging inhibition:


$$DPPH\;scavenging\;activity\;(\%\;inhibition)=[\frac{Absorbance_{control}-Absorbance_{sample}}{Absorbance_{control}}]\times 100$$

An extract concentration curve was constructed to determine the required extract concentration that causes a 50% decrease in the initial DPPH concentration. In the context of linear regression analysis, the calculated value that is obtained is referred to as IC50.

### 2.5 High-performance liquid chromatography analysis of phenolic compounds

In this study, the quantitative determination of phenolic compounds was performed using a validated HPLC-DAD method supported by accurately prepared reference standard solutions. Each phenolic reference material was first dissolved in an appropriate solvent to obtain precise stock concentrations, followed by the preparation of a six-level external calibration series at concentrations of 25, 50, 75, 100, 200, and 300 μg/mL. All calibration standards were analyzed under the same chromatographic conditions as the samples, and calibration curves were constructed by plotting detector responses against the corresponding concentrations. Linear regression analysis was applied to verify the analytical sensitivity, linearity, and reliability of quantification.

Chromatographic separation was carried out on an ACE 5 C18 reversed-phase analytical column (250 × 4.6 mm, 5 µm), which provides efficient resolution for structurally diverse phenolic compounds. A total of 14 phenolics were examined, including L-ascorbic acid; simple phenolic acids (gallic acid, 3, 4-dihydroxybenzoic acid); flavonoids [(+)-catechin, (–)-epicatechin, rutin, myricetin, quercetin, apigenin]; hydroxycinnamic acid derivatives (trans-caffeic acid, vanillic acid, ferulic acid, rosmarinic acid) and p-coumaric acid. These compounds were selected due to their biological relevance, antioxidant capacity, and frequent occurrence in plant matrices.

The mobile phase consisted of a binary gradient system of acetonitrile (A) and 1.5% (w/w) acetic acid solution (B). The gradient was initiated at 15% A and 85% B, then linearly increased to 40% A and 60% B over a total run time of 29 minutes. The flow rate was maintained at 0.7 mL/min, and the injection volume was set to 10 μL. To ensure retention time stability and reproducibility, the column temperature was controlled at 35 °C using a G7116A thermostatted column compartment.

Detection was performed using a 1260 DAD WR diode-array detector, which enabled simultaneous monitoring of phenolic compounds at 250, 270, and 320 nm based on their characteristic UV absorption maxima. The chromatographic system also included a 1260 Quaternary Pump for precise solvent delivery and a 1260 Autosampler that provided reliable and repeatable injections throughout the analytical sequence.

Overall, this comprehensive HPLC-DAD procedure ensured high-resolution separation, strong linear calibration performance, and accurate quantitative profiling of the targeted phenolic compounds within plant extracts.

### 2.6 Computational details

#### 2.6.1 Ligand preparation

The selected plant-derived antioxidants, L-ascorbic acid, gallic acid, p-coumaric acid, and rosmarinic acid, were retrieved from the PubChem database in “.sdf” format. The molecular structures were converted to “.pdb” format using Discovery Studio Visualizer. Torsional optimization was applied to evaluate the conformational flexibility of the ligands, allowing free rotation around single bonds. The optimized structures were then converted into “.pdbqt” format using the AutoDock Vina module within the PyRx platform (Trott and Olson, 2010) to ensure compatibility for docking simulations.

#### 2.6.2 Receptor preparation

The crystal structure of xanthine oxidase from Bos taurus (PDB ID: 3NVY, 2.0 Å resolution) was obtained from the RCSB Protein Data Bank (https://www.rcsb.org). This structure was selected because it represents the first mammalian xanthine oxidase complexed with the natural flavonoid inhibitor quercetin, providing a clear view of the enzyme’s active site and its interactions with phenolic scaffolds. Xanthine oxidase was also selected as an oxidative stress-related target because it is involved in reactive oxygen species generation, making it relevant to the exploratory interpretation of antioxidant-related findings. In the co-crystallized complex, quercetin is sandwiched between Phe914 and Phe1009 and forms hydrogen bonds with Arg880 and Glu802, interactions that reflect the binding behavior of structurally related phytochemicals. Before docking, all crystallographic water molecules and non-essential heteroatoms were removed. Polar hydrogens were added, and Gasteiger charges were assigned using AutoDock Tools (v4.2).

#### 2.6.3 Molecular docking and binding energy analysis

Molecular docking simulations were conducted using AutoDock Vina integrated into the PyRx software to predict the most favorable binding conformations of the selected ligands within the active site of xanthine oxidase. The docking grid box was centered on the co-crystallized quercetin-binding region of the 3NVY structure to accurately encompass the catalytic pocket surrounding the molybdenum cofactor. Among the generated poses, the complexes with the lowest binding free energy (kcal/mol) were selected for further analysis. The binding modes and key molecular interactions, including hydrogen bonds, π–π stacking, and hydrophobic contacts, were visualized and analyzed using Discovery Studio Visualizer.

### 2.7 Statistical analysis

All data were statistically processed in the StatSoft Inc. program, STATISTICA, version 8.0 (Tulsa, OK, USA). Statistical processing of the data included analysis of variance using a one-way ANOVA and separation based on Fisher’s LSD test at a significance level of p ≤ 0.05. A graphic presentation of the results was done using the computer program Microsoft Office Excel (Redmond, WA, USA).

## 3 Results

### 3.1 Morphological traits of pea sprouts and microgreens

The FW and %DW of sprouts showed significant differences under different LED lights treatments (**Table 1**). The highest FW was recorded under blue-red light (BR) treatment (0.169 g), followed by the cool white (CW) (0.140 g), the blue (B) (0.129 g), the dark (D) (0.123 g) and the red (R) light treatment (0.113 g). BR treatment increased sprouts FW by 49.6% compared to R light and by 37.4% compared to darkness. This indicates that mixed spectra enhanced early biomass growth more than monochromatic light or darkness. The highest percentage of DW was under CW (10.20%), followed by B (9.63%) and BR treatment (9.55%). The lowest %DW was recorded under red light (9.24%) and darkness (8.92%). The percentage increase in %DW under CW compared to D was approximately 14.4%. This indicates that the tissue structure was thicker and a greater amount of solid matter was collected under full-spectrum lighting. The BR treatment yielded the most ADM (0.0161 g), followed by CW (0.0142 g), B (0.0124 g), D (0.0109 g), and R (0.0104 g).

**Table 1:** Morphological growth parameters of pea sprouts.

Light treatment	Stem FW (g) ± SD*	Stem DW (%) ± SD	ADM (g)
CW **	0.140 ± 0.015 b	10.197 ± 0.373 a	0.0142 ± 0.0009 a
B	0.129 ± 0.020 bc	9.633 ± 0.309 b	0.0124 ± 0.0004 b
R	0.113 ± 0.016 c	9.243 ± 0.147 b	0.0104 ± 0.0005 b
BR	0.169 ± 0.031 a	9.551 ± 0.255 b	0.0161 ± 0.0014 a
D	0.123 ± 0.024 bc	8.917 ± 0.220 c	0.0109 ± 0.0012 b

*FW, fresh weight; DW (%), dry weight (%); ADM-Absolute dry mass; SD, standard deviation. **CW, cool white; B, blue; R, red; BR, blue:red 1:1; D, darkness. Results are expressed as a mean value ± SD (n=10). Means with the same small letters within the same column, for sprouts and microgreens separetely, are not significantly different (p < 0.05).

The light treatments had a significant effect on the growth characteristics of pea microgreens, including FW, %DW and length (**Table 2**). The highest FW was found in D conditions (0.482 g), followed by B (0.291 g), R (0.282 g), CW (0.267 g) and the lowest in BR (0.263 g). The highest %DW values were recorded under CW light (11.65%), followed by BR (11.21%) and B (11.09%), while the lowest values were observed under R (9.89%) and D (7.41%) treatments. When we combined FW and %DW to calculate the absolute dry mass the D treatment still yielded the highest value (0.0358 g), followed by B (0.0323 g), CW (0.0311 g), BR (0.0295 g), and R (0.0279 g). The high ADM observed in darkness is mostly due to the increase in FW due to water uptake and elongation, rather than an improvement in the plant’s physiological quality. Photosynthetically active light treatments, on the other hand, lead to lower biomass but more organized tissue structures.

**Table 2:** Morphological growth parameters of pea microgreens.

Light treatment	Stem FW (g) ± SD*	Stem DW (%) ± SD	ADM (g)	Stem length (mm)
CW**	0.267 ± 0.035 b	11.651 ± 0.410 a	0.031 ± 0.002 ab	71.00 ± 6.73 c
B	0.291 ± 0.036 b	11.095 ± 0.671 a	0.032 ± 0.001 ab	92.44 ± 5.46 b
R	0.282 ± 0.084 b	9.888 ± 0.205 b	0.027 ± 0.007 b	91.22 ± 12.10 b
BR	0.263 ± 0.037 b	11.214 ± 0.236 a	0.029 ± 0.002 ab	68.89 ± 7.72 c
D	0.482 ± 0.070 a	7.414 ± 0.632 c	0.036 ± 0.004 a	202.89 ± 21.89 a

*FW− fresh weight; DW (%) − dry weight (%); ADM-Absolute dry mass; SD – standard deviation. ** CW – cool white, B – blue, R – red, BR – blue:red 1:1; D – darkness. Results are expressed as a mean value ± SD (n=10). Means with the same small letters within the same column, for sprouts and microgreens separetely, are not significantly different (p < 0.05).

Length measurements revealed that the morphology underwent significant changes in response to different light spectra. The longest elongation happened in the dark (202.89 mm), followed by B (92.44 mm) and R (91.22 mm). Under CW (71.00 mm) and BR treatment (68.89 mm) plants were shorter and more compact. In darkness microgreens were characterized by greater elongation and a lower percentage of dry weight (DW), while in CW and BR conditions their elongation was better controlled.

### 3.2 Pigment composition under LED treatments

The accumulation of photosynthetic pigments in pea sprouts and microgreens was significantly affected by the applied LED treatment (**Table 3**, **Table 4**). In sprouts the highest chlorophyll a concentration was observed under BR light (0.741 mg/g DW), followed by R (0.528 mg/g DW) and CW (0.449 mg/g DW). B light produced a lower chlorophyll a content (0.381 mg/g DW), while D resulted in the lowest value (0.134 mg/g DW). Chlorophyll b levels remained low but followed a similar trend, with BR treatment yielding the highest value (0.171 mg/g DW) and D the lowest (0.028 mg/g DW). Total chlorophyll (Chl a+b) was maximized under BR light (0.912 mg/g DW) and was comparable between R and CW treatments (0.661 and 0.576 mg/g DW, respectively), but lower under B and especially D conditions. Carotenoid accumulation was greatest under B (0.235 mg/g DW) and BR light (0.268 mg/g DW), followed by R and CW treatments (0.197 and 0.182 mg/g DW). The lowest carotenoid content was observed in darkness (0.069 mg/g DW).

**Table 3:** The total content of phytochemical compounds in pea sprouts extract.

Light treatment	Chl a (mg/g DW) ± SD*	Chl b (mg/g DW) ± SD	Chl a+b (mg/g DW) ± SD	TCC (mg/g DW) ± SD	TPC (mg GAE/g DW) ± SD	TFC (mg QE/g DW) ± SD	DPPH (IC50 mg/mL) ± SD
CW**	0. 449 ± 0.088 b	0.126 ± 0.032 b	0.576 ± 0.120 b	0.182 ± 0.036 b	3.435 ± 1.381 c	0.883 ± 0.001 c	2.538 ± 0.149 c
**B**	0.381 ± 0. 089 b	0.078 ± 0.006 b	0.459 ± 0.083 b	0.235 ± 0.037 a	5.869 ± 0.281a	0.929 ± 0.107 bc	2.561 ± 0.159 c
**R**	0.528 ± 0.088 b	0.132 ± 0.026 b	0.661 ± 0.115 b	0.197 ± 0.020 b	3.713 ± 0.467 c	1.027 ± 0.033 ab	2.863 ± 0.008 a
**BR**	0.741 ± 0.104 a	0.171 ± 0.016 a	0.912 ± 0.120 a	0.268 ± 0.034 a	4.992 ± 0.105 ab	1.046 ± 0.145 a	2.629 ± 0.155 bc
**D**	0.134 ± 0.021 c	0.028 ± 0.002 c	0.162 ± 0.018 c	0.069 ± 0.002 c	4.209 ± 0.371 bc	0.952 ± 0.064 bc	2.807 ± 0.153 ab

*DW, dry weight; Chl a+b, total chlorophyll content; TCC, total carotenoid content; TPC, total phenolic content; TFC, total flavonoid content; DPPH, total antioxidative capacity; GAE gallic acid equivalents; QE, quercetin equivalents; SD, standard deviation. **CW, cool white; B, blue; R, red; BR, blue:red 1:1; D, darkness. Results are expressed as a mean value ± SD (n=3). Means with the same small letters within the same column are not significantly different (*p*  < 0.05).

**Table 4:** The total content of phytochemical compounds in pea microgreens extract.

Light treatment	Chl a (mg/g DW) ± SD*	Chl b (mg/g DW) ± SD	Chl a+b (mg/g DW) ± SD	TCC (mg/g DW) ± SD	TPC (mg GAE/g DW) ± SD	TFC (mg QE/g DW) ± SD	DPPH (IC50 mg/mL) ± SD
CW**	5.154 ± 0.413 ab	2.547 ± 0.079 ab	7.701 ± 0.334 ab	1.189 ± 0.180 ab	7.531 ± 0.522 a	1.307 ± 0.212 b	2.534 ± 0.108 b
B	5.849 ± 1.295 a	1.861 ± 0.407 b	7.710 ± 1.693 ab	1.454 ± 0.350 a	6.243 ± 0.748 ab	1.403 ± 0.341 ab	2.669 ± 0.054 b
R	4.169 ± 0.393 b	2.489 ± 0.836 ab	6.658 ± 0.673 b	0.799 ± 0.367 b	6.081 ± 0.558 ab	1.406 ± 0.033 ab	2.871 ± 0.299 a
BR	6.139 ± 1.058 a	3.117 ± 0.732 a	9.257 ± 1.429 a	1.301 ± 0.326 ab	5.861 ± 0.336 ab	1.759 ± 0.303 a	2.615 ± 0.258 b
D	1.441 ± 0.078 c	0.436 ± 0.022 c	1.301 ± 0.326 c	0.391 ± 0.036 c	4.679 ± 0.492 b	0.881 ± 0.023 c	3.025 ± 0.049 a

*DW, dry weight; Chl a+b, total chlorophyll content; TCC, total carotenoid content; TPC, total phenolic content; TFC, total flavonoid content; DPPH, total antioxidative capacity; GAE gallic acid equivalents; QE, quercetin equivalents; SD, standard deviation. **CW, cool white; B, blue; R, red; BR, blue:red 1:1; D, darkness. Results are expressed as a mean value ± SD (n=3). Means with the same small letters within the same column are not significantly different (*p*  < 0.05).

The highest chlorophyll a concentration (6.139 mg/g DW) was recorded under the combined BR light treatment, followed closely by B (5.849 mg/g DW) and CW light (5.154 mg/g DW) at microgreens. The R light treatment resulted in a lower chlorophyll a value (4.169 mg/g DW), whereas the D treatment showed the lowest pigment accumulation (1.441 mg/g DW). Chlorophyll b content followed a similar pattern: BR treatment produced the highest value (3.117 mg/g DW), while the lowest was observed under D conditions (0.436 mg/g DW). Total chlorophyll (Chl a+b) was significantly enhanced by BR light (9.257 mg/g DW), exceeding all other treatments. CW and B light treatments induced intermediate levels (7.701 and 7.710 mg/g DW respectively), whereas R and D treatments resulted in significantly lower totals (6.658 and 1.301 mg/gDW, respectively). Carotenoid content was highest under B light (1.454 mg/g DW), followed by BR (1.301 mg/g DW) and CW light (1.189 mg/g DW). R light resulted in moderate accumulation (0.799mg/gDW), while D conditions yielded the lowest carotenoid concentration (0.391 mg/g DW).

In all treatments, microgreens had significantly higher pigment concentrations than sprouts, reflecting their more advanced photosynthetic capacity (**Table 4**). Chlorophyll a and total chlorophyll (a+b) reached their highest levels under combined BR light in both developmental stages, with absolute concentrations in microgreens 10 to12 times higher than in sprouts. For example, total chlorophyll under BR light increased from 0.912 mg/g DW in sprouts to 9.257 mg/g DW in microgreens. Carotenoid levels were also consistently higher in microgreens, with the highest values observed under B (1.454 mg/g DW) and BR (1.301 mg/g DW) light. In contrast, sprouts showed modest carotenoid accumulation under B (0.235 mg/g DW) and BR (0.268 mg/g DW) light, with a marked decrease under dark conditions. Dark-grown samples had the lowest pigment values in both developmental stages, confirming that light is essential for the induction of chlorophyll and carotenoid biosynthesis.

### 3.3 Antioxidant potential assessment

The total phenolic content (TPC) in pea sprouts and microgreens varied significantly across the different LED light treatments (**Tables 3**, **4**). In sprouts, the highest TPC was recorded under blue light (5.869 mg GAE/g DW), which was significantly greater than under cool white (3.435 mg GAE/g DW) or red (3.713 mg GAE/g DW) lights, and higher than under the combined blue-red (4.992 mg GAE/g DW) or dark (4.209 mg GAE/g DW) conditions, with statistical groupings indicating distinct differences. This suggests that blue wavelengths, particularly, enhance phenolic biosynthesis during early growth stages, potentially through the activation of photoreceptors linked to secondary metabolism, while mixed or absent light yields intermediate or lower accumulation. In microgreens, the highest TPC was observed under CW light (7.531 mg GAE/g DW), which was significantly greater than under D (4.679 mg GAE/g DW) conditions, and comparable to B (6.243 mg GAE/g DW), R (6.081 mg GAE/g DW), and combined BR (5.861 mg GAE/g DW) lights, with statistical differences determined by Fisher’s LSD test (p ≤ 0.05). This suggests that broad-spectrum white light promotes higher phenolic biosynthesis in more mature stages, likely through comprehensive photoreceptor stimulation that supports secondary metabolism, whereas monochromatic or absent light results in intermediate or reduced accumulation.

The total flavonoid content (TFC) in pea sprouts varied significantly across the different LED light treatments (**Table 3**). The highest TFC was recorded under the combined BR light (1.046 mg QE/g DW), which was significantly greater than under CW (0.883 mg QE/g DW), B (0.929 mg QE/g DW), or D (0.952 mg QE/g DW) conditions, and comparable to R (1.027 mg QE/g DW), with statistical groupings indicating distinct differences based on Fisher’s LSD test (p ≤ 0.05). Notably, the lowest TFC was observed under CW light, while darkness yielded intermediate levels rather than the minimum. These reuslts suggest that synergistic red and blue wavelengths particularly enhance flavonoid biosynthesis during early growth stages, potentially through upregulation of phenylpropanoid pathway enzymes. Conversely, broad-spectrum white light suppresses accumulation, while monochromatic or absent light results in moderate to higher levels.

In microgreens, the highest TFC was observed under combined BR light (1.759 mg QE/g DW), which was significantly greater than under CW (1.307 mg QE/g DW) or D (0.881 mg QE/g DW) conditions, and comparable to B (1.403 mg QE/g DW) and R (1.406 mg QE/g DW), with statistical differences determined by Fisher’s LSD test (p ≤ 0.05). This suggests that synergistic red and blue wavelengths promote superior flavonoid biosynthesis in more mature stages, potentially through the upregulation of light-responsive pathways. In contrast, broad-spectrum white or absent light yields reduced accumulation, while monochromatic lights result in intermediate levels.

Comparatively, microgreens exhibited consistently elevated TPC and TFC than sprouts across treatments, highlighting a developmental shift toward enhanced secondary metabolite production, nutritional quality and antioxidant potential. However, darkness suppressed TFC levels to values similar to those measured in sprouts (**Table 3**).

The antioxidant capacity, assessed via DPPH radical scavenging activity (expressed as IC50 in mg/mL), in pea sprouts exhibited significant overall variation across the LED light treatments (**Table 3**), though differences between certain groups were not pronounced. The lowest IC50 values, indicating stronger antioxidant potential, were observed under CW (2.538 mg/mL) and B (2.561 mg/mL) lights, which were statistically comparable to each other and to combined BR (2.629 mg/mL), but significantly lower than under R (2.863 mg/mL) light. Dark (2.807 mg/mL) conditions yielded intermediate values, similar to R and partially overlapping with BR, as determined by Fisher’s LSD test (p ≤ 0.05). This pattern suggests that while broad-spectrum white and blue-enriched lights modestly enhance early-stage radical scavenging efficiency, potentially via balanced photoreceptor activation and subtle boosts in antioxidant metabolite production, the effects are not dramatically differentiated among CW, B, and BR, nor between R and D, implying a limited sensitivity of DPPH activity to spectral nuances in sprouts under these experimental conditions.

In microgreens, the antioxidant capacity, assessed via DPPH radical scavenging activity (expressed as IC50 in mg/mL), exhibited significant overall variation across the LED light treatments (**Table 4**), though differences between certain groups were not pronounced. The lowest IC50 values, indicating stronger antioxidant potential, were observed under CW (2.534 mg/mL), combined BR (2.615 mg/mL), and B (2.669 mg/mL) lights, which were statistically comparable to each other but significantly lower than under R (2.871 mg/mL) light or D (3.025 mg/mL) conditions, where R and D showed similar higher values, as determined by Fisher’s LSD test (p ≤ 0.05). This pattern suggests that while broad-spectrum white, blue-enriched, and mixed lights modestly enhance radical scavenging efficiency in more mature stages. This effect may be modulated through subtle boosts in antioxidant metabolites. However, the effects are not dramatically differentiated among CW, BR, and B, nor between R and D (with BR partially overlapping broader ranges), indicating a limited sensitivity of DPPH activity to spectral nuances in microgreens under these experimental conditions. Comparatively, IC50 values in microgreens were broadly similar to those in sprouts, indicating consistent antioxidant responses across developmental stages, albeit with light-dependent modulations (**Tables 3**, **4**).

### 3.4 Polyphenolic profile analysis

In this study, the concentrations of phenolic compounds and L-ascorbic acid were quantitatively determined for sprouts grown under different LED light treatments (CW, B, R, BR, and D) using the HPLC-DAD analytical method (**Table 5**). Among vitamins, only L-ascorbic acid was detected, with concentrations ranging from 256.49 mg/L to 709.24 mg/L, indicating substantial variation among sample sets. Within the phenolic acids, gallic acid was consistently present in all groups and exhibited relatively high concentrations, ranging from 258.38 mg/L to 598.70 mg/L. p-Coumaric acid was detectable in selected sample groups and showed particularly elevated levels in sprouts grown under B (231.92 mg/L) and BR light combined treatment (324.73 mg/L). Rosmarinic acid was detected in all groups at low but stable concentrations, ranging from 14.00 mg/L to 19.13 mg/L.

**Table 5:** Concentration profiles of detected bioactive compounds in sprouts.

LED treatment compound (mg/L) + SD	CW*	B	R	BR	D
	Vitamin				
L-Ascorbic acid	256.49 ± 5.04	709.24 ± 7.40	454.50 ± 9.19	519.00 ± 31.11	524.00 ± 15.56
Phenolics					
Gallic acid	478.33 ± 8.10	258.38 ± 11.77	569.75 ± 5.87	550.13 ± 26.12	598.70 ± 25.60
3, 4-Dihydroxybenzoic acid	N/D**	N/D	N/D	N/D	N/D
Vanillic acid	N/D	N/D	N/D	N/D	N/D
p-Coumaric Acid	43.64 ± 1.12	231.92 ± 5.47	N/D	324.73 ± 7.13	N/D
Trans-Caffeic acid	N/D	N/D	N/D	N/D	N/D
Ferulic acid	N/D	N/D	N/D	N/D	N/D
Rosmarinic acid	19.13 ± 4.94	15.36 ± 0.69	15.69 ± 2.14	14.00 ± 1.41	15.77 ± 0.11
Flavonoids					
(+)-Catechin	422.86 ± 7.71	504.44 ± 10.98	634.50 ± 17.10	N/D	57.20 ± 3.46
(-)-Epicatechin	254.25 ± 17.04	696.08 ± 12.13	72.02 ± 1.08	511.15 ± 5.59	22.24 ± 3.71
Rutin	N/D	N/D	N/D	N/D	N/D
Myricetin	N/D	N/D	N/D	N/D	N/D
Quercetin	N/D	N/D	81.34 ± 0.08	N/D	N/D
Apigenin	N/D	N/D	N/D	N/D	N/D

*CW, cool white; B, blue; R, red; BR, blue:red 1:1; D, darkness. **N/D, No data / Not determined.

Among the flavonoids, (+)-catechin exhibited the highest concentration in the sprouts grown under R light (634.50 mg/L), while (–)-epicatechin reached its maximum in those grown under B light (696.08 mg/L). Quercetin was detected only in the R light treatment group at a concentration of 81.34 mg/L. Several phenolic compounds, including 3, 4-dihydroxybenzoic acid, vanillic acid, trans-caffeic acid, ferulic acid, rutin, myricetin, and apigenin, were not detected in any of the sample groups (N/D). Overall, the results demonstrate notable variability in phenolic profiles among the sample sets, with gallic acid, catechin, and epicatechin emerging as the predominant phenolic constituents.

The concentrations of L-ascorbic acid and various phenolic compounds were evaluated in microgreens sample groups using the HPLC-DAD analytical system (**Table 6**). L-ascorbic acid was consistently detected in all groups at comparatively high levels, ranging from 389.00 mg/L to 1178.51 mg/L. Among phenolic acids, gallic acid was present in all sample sets and exhibited stable concentrations ranging from 113.10 mg/L to 139.59 mg/L. The compound 3, 4-dihydroxybenzoic acid was quantifiable only in select groups, reaching its highest concentration in microgreens grown under dark conditions (52.75 mg/L). Vanillic acid was detected at a very low concentration exclusively in the darkness group (1.57 mg/L). p-Coumaric acid displayed pronounced variation across sample groups, with markedly high levels in microgreens grown under CW (1129.75 mg/L) and B light (830.34 mg/L), decreasing substantially under R light (265.56 mg/L) and showing a wide variance range under BR (585.25 to 678.18 mg/L), while remaining undetected in darkness (D). Rosmarinic acid was present in all groups at low but consistent concentrations ranging from 14.19 mg/L to 22.73 mg/L.

**Table 6:** Concentration profiles of detected bioactive compounds in microgreens.

LED treatment compound (mg/L) + SD	CW*	B	R	BR	D
	Vitamin				
L-Ascorbic acid	1178.51 ± 10.46	1127.80 ± 51.98	904.51 ± 4.45	845.71 ± 0.18	389.00 ± 7.07
Phenolics					
Gallic acid	139.59 ± 0.40	127.75 ± 11.53	113.10 ± 0.42	128.80 ± 2.83	124.10 ± 2.12
3, 4-Dihydroxybenzoic acid	N/D	7.17 ± 0.11	6.30 ± 0.42	N/D	52.75 ± 3.89
Vanillic acid	N/D	N/D	N/D	N/D	1.57 ± 0.11
p-Coumaric Acid	1129.75 ± 18.60	830.34 ± 18.22	265.56 ± 8.53	585.25 ± 678.18	N/D
Trans-Caffeic acid	N/D	N/D	N/D	N/D	N/D
Ferulic acid	N/D	N/D	N/D	N/D	N/D
Rosmarinic acid	22.60 ± 0.00	20.43 ± 0.95	21.01 ± 0.48	22.73 ± 0.18	14.19 ± 0.16
Flavonoids					
(+)-Catechin	N/D	1464.97 ± 31.43	N/D	N/D	N/D
(-)-Epicatechin	1427.50 ± 21.78	872.62 ± 14.84	712.22 ± 4.88	1132.27 ± 63.78	N/D
Rutin	N/D	N/D	N/D	N/D	N/D
Myricetin	N/D	N/D	N/D	N/D	N/D
Quercetin	82.90 ± 0.28	82.46 ± 0.01	82.05 ± 0.26	82.28 ± 0.42	82.45 ± 0.35
Apigenin	N/D	N/D	N/D	N/D	N/D

*CW, cool white; B, blue; R, red; BR, blue:red 1:1; D, darkness. **N/D, No data / Not determined.

Within the flavonoid class, (+)-catechin was detected only in the blue light treatment group at a notably high concentration (1464.97 mg/L). (–)-Epicatechin, however, was quantified at elevated levels in CW, B, R, and BR light treatment groups, with concentrations ranging between 712.22 mg/L and 1427.50 mg/L but was absent in the D group. Quercetin demonstrated remarkable stability across all sample groups, consistently measured within a narrow interval of approximately 82 mg/L (82.05 – 82.90 mg/L). Other phenolics, including trans-caffeic acid, ferulic acid, rutin, myricetin, and apigenin, were not detected in any of the examined groups (N/D). Collectively, these findings indicate substantial compositional variability among the evaluated sample sets, with L-ascorbic acid, gallic acid, p-coumaric acid, (–)-epicatechin, and quercetin emerging as the predominant compounds within microgreens samples.

### 3.5 Molecular docking results

The molecular docking analysis performed on xanthine oxidase (PDB ID: 3NVY) provided a comparative assessment of the predicted binding behavior of different plant-derived antioxidant compounds toward the enzyme. Among the evaluated ligands, rosmarinic acid showed the most favorable predicted binding affinity (−9.2 kcal/mol) and the lowest estimated inhibition constant (Ki = 0.178 µM). Similarly, catechin (−9.1 kcal/mol, Ki = 0.211 µM) and epicatechin (−8.3 kcal/mol, Ki = 0.815 µM) also demonstrated relatively strong predicted binding affinities and low Ki values.

Other phenolic compounds, including gallic acid (−6.5 kcal/mol), p-coumaric acid (−6.4 kcal/mol), and L-ascorbic acid (−6.3 kcal/mol), demonstrated moderate binding energies, indicating relatively weaker inhibitory activity. Rosmarinic acid also displayed superior Fit Quality (FQ = 0.843) and an appropriate Binding Efficiency Index (BEI = 0.026), highlighting its excellent structural complementarity and energetic efficiency (**Table 7**).

**Table 7:** Molecular docking results of plant-derived antioxidant compounds showing binding affinities, ligand efficiency (LE), fit quality (FQ), binding efficiency index (BEI), and estimated inhibition constants (Ki) against xanthine oxidase (PDB ID: 3NVY).

Compound	Binding energy (kcal/mol)	LE^*^	FQ	BEI	Ki (μM)
L-Ascorbic acid	-6.3	0.525	0.483	0.036	23.894
Gallic acid	-6.5	0.542	0.499	0.038	17.044
p-coumaric acid	-6.4	0.533	0.491	0.039	20.180
Rosmarinic acid	-9.2	0.354	0.843	0.026	0.178
Catechin	-9.1	0.433	0.822	0.031	0.211
Epicatechin	-8.3	0.395	0.749	0.029	0.815

*LE, Ligand Efficiency; FQ, Fit Quality; BEI, Binding Efficiency Index; Ki, Estimated Inhibition Constant.

**Figure 1:**
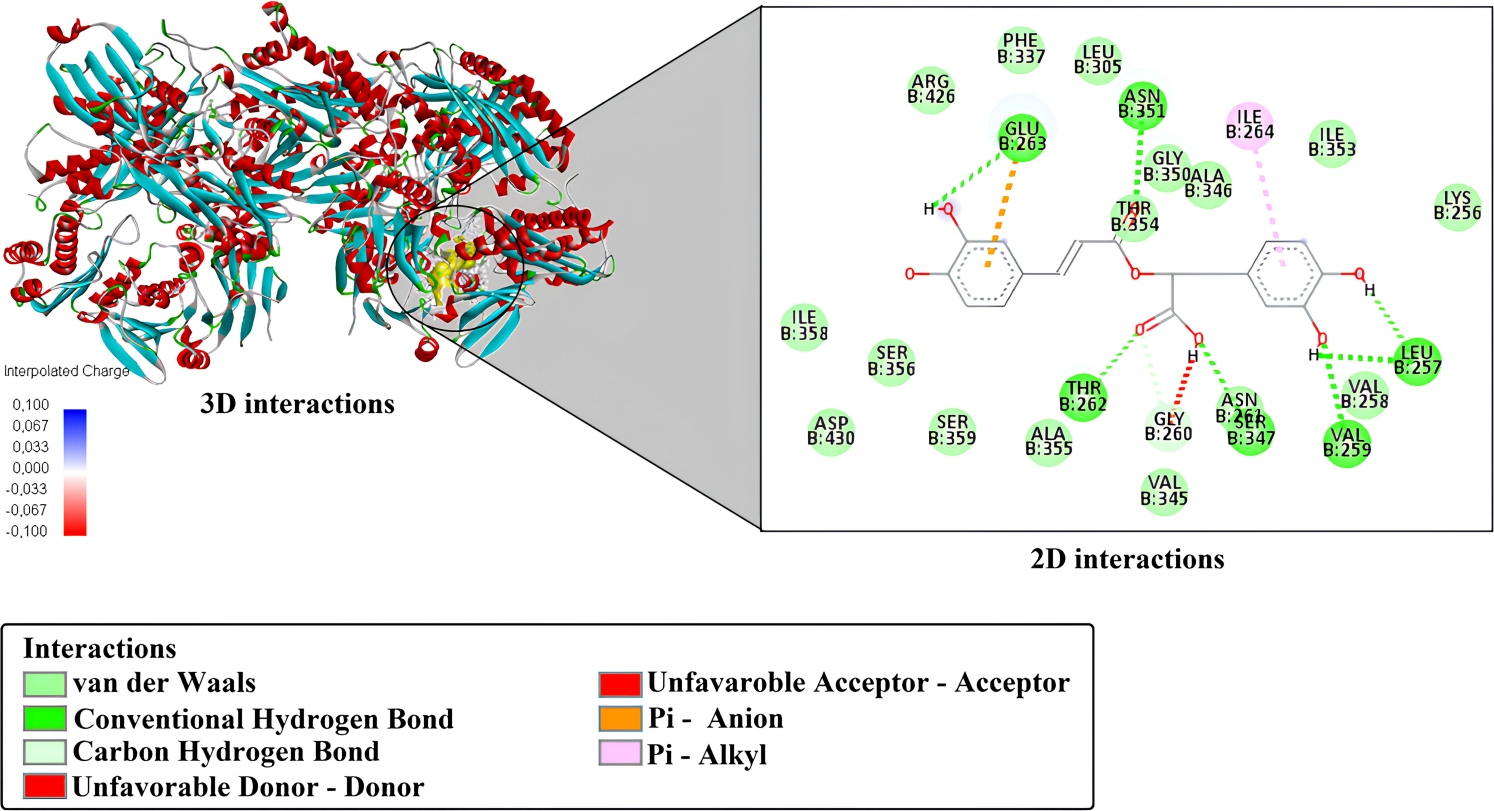
Three-dimensional and two-dimensional interaction diagrams of rosmarinic acid within the active site of xanthine oxidase (PDB ID: 3NVY). The 3D representation (left) shows the spatial orientation of the ligand in the catalytic pocket, while the 2D interaction map (right) illustrates key molecular interactions, including conventional hydrogen bonds, van der Waals forces, π–anion, and π–alkyl interactions with critical amino acid residues.

The molecular interaction analysis revealed that both rosmarinic acid and catechin established multiple stabilizing interactions within the active site of xanthine oxidase (PDB ID: 3NVY) (**Table 8**). Rosmarinic acid formed an extensive hydrogen-bonding network with key residues including LEU257, THR262, GLY260, GLU263, SER347, and ASN351, indicating strong electrostatic stabilization within the catalytic pocket. The ligand also engaged in a π–alkyl interaction with ILE264, enhancing hydrophobic stabilization. No π–π stacking interaction was detected, suggesting that the binding affinity of rosmarinic acid is primarily governed by hydrogen bonding and van der Waals forces (**Figure 1**).

**Table 8:** Hydrogen bond, π–π stacking, and alkyl interaction profiles of rosmarinic acid and catechin within the active site of xanthine oxidase.

Compounds	H-bond	Pi–Pi stacking	Alkyl interactions
Rosmarinic acid	AL259:HN–O2 THR262:HN–O5 SER347:HN–O4 ASN351:HN–O6 H1–LEU257:O H2–LEU257:O H10–GLU263:OE1 GLY260:HA1–O5 GLU263:OE1–	–	ILE264 (π–Alkyl)
Catechin	VAL259:HN–O4 H1–THR262:OG1 H3–LEU257:O H9–ASN351:OD1 H10–ASN351:OD1 (Conventional H-Bonds) GLY260:HA1–O2 GLY350:HA2–O1 (C–H Bonds)	GLY350:C, O;ASN351:N–Ligand (Amide–π Stacked)	ILE264 (π–Alkyl) ALA346 (π–Alkyl)

**Figure 2:**
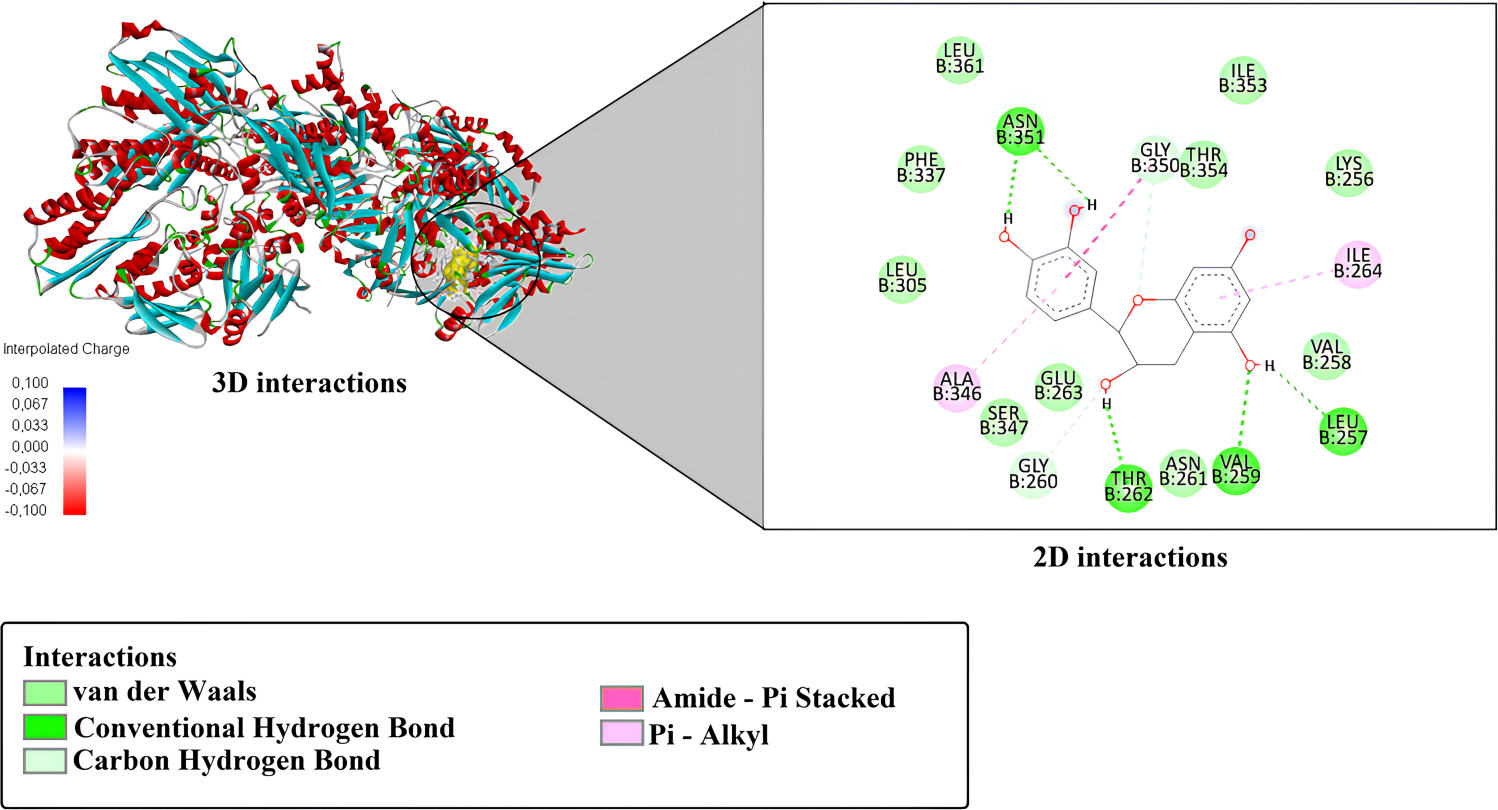
Three-dimensional and two-dimensional interaction diagrams of catechin within the active site of xanthine oxidase (PDB ID: 3NVY). The 3D representation (left) shows the spatial orientation of the ligand inside the catalytic pocket, while the 2D interaction map (right) illustrates the major molecular interactions, including conventional hydrogen bonds, amide–π stacking, and π–alkyl interactions with key amino acid residues.

In contrast, catechin displayed a combination of conventional hydrogen bonds with VAL259, THR262, LEU257, and ASN351, as well as C–H interactions with GLY260 and GLY350, contributing to its stable orientation in the binding site. Moreover, an amide–π stacking interaction between ASN351 and the aromatic ring of catechin was observed, accompanied by π–alkyl interactions with ILE264 and ALA346, further reinforcing hydrophobic contacts within the pocket (**Figure 2**). Overall, both ligands showed favorable predicted interactions with the enzyme through a combination of hydrogen bonds and hydrophobic contacts. However, the higher number and greater diversity of polar interactions observed for rosmarinic acid may provide a structural basis for its more favorable predicted binding energy compared with catechin.

## 4 Discussion

The study demonstrates that light quality significantly affects growth and physiological traits in sprouts and microgreens, though sprout responses should be interpreted cautiously due to their predominantly heterotrophic stage, where metabolism is driven by seed reserves. The joint analysis captures the transition toward light-regulated metabolism rather than implying equivalent responses between stages.

In sprouts, blue and red light (BR) increased fresh weight (FW), while monochromatic red (R) reduced FW and percentage dry weight (%DW), promoting stem elongation without thickening due to the absence of blue light (Snowden et al., 2016). As sprouts are largely heterotrophic, FW increases mainly reflect water uptake and cell elongation rather than true biomass, and LED responses are associated with elongation dynamics and tissue hydration rather than photosynthesis (Paradiso and Proietti, 2022). In contrast, microgreens are photosynthetically active, and light quality regulates growth, biomass allocation, and tissue organization. Cool white (CW) and BR lighting produced shorter plants with higher %DW, indicating greater compactness. CW supports photosynthesis by combining blue, red, and green wavelengths (Kyriacou et al., 2019a; Ouzounis et al., 2015; Pennisi et al., 2019), while BR improves tissue structure and biomass (Paucek et al., 2020; Brazaitytė et al., 2021). Blue light alone resulted in moderate elongation and %DW but was less effective without red light (Meng et al., 2019), consistent with salad, kale, and amaranth microgreens (Brazaitytė et al., 2021). Red light produced longer, thinner stems, while darkness (D) induced etiolation with high FW but low %DW, reflecting weak tissues (Kyriacou et al., 2019a; Snowden et al., 2016). These results align with studies showing mixed spectra (CW, BR) improve biomass, pigments, and compactness, whereas darkness promotes etiolation and poor quality (Paradiso and Proietti, 2022; Meng et al., 2019, Meng et al., 2020). In pea microgreens, CW and BR appear optimal for growth and quality. Microgreens showed higher FW and %DW than sprouts across treatments, increasing under CW and darkness. Their longer growth period and functional chloroplasts enable greater dry matter accumulation (Kyriacou et al., 2019a; Meng et al., 2019; Bantis et al., 2018). However, darkness still produced high FW (0.482 g) but low %DW (7.41%), indicating etiolation (Snowden et al., 2016). While FW may overestimate growth under some conditions, %DW is a more reliable indicator of quality and biomass, especially across developmental stages. These findings agree with studies on lettuce, kale, and amaranth microgreens (Kyriacou et al., 2019a; Paucek et al., 2020; Pennisi et al., 2019).

The quality of light and the stage of development influenced the accumulation of pigment. The highest chlorophyll levels were recorded in sprouts and microgreens under BR light, reflecting the combined effect of blue light on chlorophyll synthesis and red light on photosynthesis intensity (Brazaitytė et al., 2021). This synergy has been observed in various microgreen crops, including lettuce, broccoli, cabbage and radish (Lee et al., 2016; Demir et al., 2023). In cauliflower microgreens, the highest pigment content was observed when the blue-to-red light ratio was 2:1 (Pavlović et al., 2024). Only blue light affected carotenoid accumulation, consistent with its role in activating photoprotective mechanisms (Bantis et al., 2018). In darkness, pigment levels were extremely low, particularly in sprouts, due to the limited synthesis of chlorophyll under these conditions (Mastropasqua et al., 2020). Although light can stimulate pigment synthesis in sprouts, this remains limited and transitional, reflecting early chloroplast development rather than fully functional photosynthesis. Pigment responses at this stage represent incipient photomorphogenic adjustments rather than true photosynthetic performance. Microgreens showed higher pigment content under all treatments due to functional chloroplasts and active photosynthesis. For instance, total chlorophyll increased from 0.912 to 9.257 mg/g DW under BR light, reflecting the transition from heterotrophic to photoautotrophic growth, chloroplast differentiation, and increased photosynthetic capacity (Wojdylo et al., 2020). Blue light enhanced carotenoid accumulation in both stages via photoreceptor activation, although levels in sprouts remained low (Bantis et al., 2018). Overall, light quality more strongly influences pigment biosynthesis in microgreens, where the photosynthetic apparatus is fully developed. BR light was most effective, increasing total chlorophyll 12-fold due to synergistic red–blue effects (Lee et al., 2016). This agrees with studies showing mixed spectra enhance pigment accumulation and quality in microgreens compared to monochromatic or dark conditions (Kyriacou et al., 2019a; Bian et al., 2014; Pennisi et al., 2019). However, pigment accumulation can be greater under red light depending on species, light intensity, and photoperiod (Marques et al., 2021), indicating the optimal spectrum depends on developmental stage and species. The relatively low red photon contribution in the cool white treatment (~10–15%) compared to monochromatic red LED likely contributed to reduced photosynthetic efficiency but improved light penetration due to the higher proportion of green wavelengths.

Heterotrophic sprouts, traditionally considered unresponsive to light, can react to LED light using photoreceptors to absorb LED light as a developmental signal rather than as an energy source until chlorophyll synthesis starts (Mlinarić et al., 2020; Pipitone et al., 2021; Paradiso and Proietti, 2022). Brief exposure to LED light triggers pigment biosynthesis, induces secondary metabolites (TPC, TFC), and increases antioxidant activity (DPPH) (Nam et al., 2018; Vitale et al., 2021). Significant variations in total phenolic content (TPC) were observed in pea sprouts and microgreens in response to different LED light spectra, highlighting the importance of light quality in modulating the biosynthesis of secondary metabolites. Since the sprouts are heterotrophic, this response likely reflects early activation of light-sensitive signaling pathways and enzymes in the phenylpropanoid pathways, while in microgreens, light treatment exerts more direct and physiologically meaningful effects (Avezum et al., 2023; Artés-Hernández et al., 2022; Rahman et al., 2021). Blue LED light has been found to be particularly effective in increasing the total phenolic content in several types of sprouts (Hossen, 2007; Park et al., 2020). For instance, in soybean sprouts, treatment with blue LED resulted in a higher total phenolic content compared to control conditions (Azad et al., 2018). The elevated TPC in sprouts under B light (5.869 mg GAE/g DW) compared to other treatments is consistent with previous findings that blue wavelengths stimulate phenolic accumulation during the early stages of germination, potentially by activating cryptochrome photoreceptors that increase the activity of enzymes in the phenylpropanoid pathway, such as phenylalanine ammonia-lyase (PAL) (Liu et al., 2016; Qian et al., 2016). This finding is in accordance with the documented evidence of a 25% increase in the TPC of pea sprouts under blue LED exposure in comparison to darkness or other spectra, a phenomenon attributed to an enhanced stress response and antioxidant defense mechanisms (Liu et al., 2016). The intermediate TPC in combined BR and darkness D conditions in our study suggests a synergistic but not maximal effect of mixed spectra, while the lower values under R and CW may reflect suboptimal activation of phenolic-specific pathways.

The total phenolic content (TPC) in pea microgreens exhibited significant variations in response to different LED light spectra, emphasizing the impact of light quality on phenolic biosynthesis during advanced developmental stages. The superior TPC under CW light (7.531 mg GAE/g DW) was found to be significantly higher than in D conditions (4.679 mg GAE/g DW) and comparable to B, R, and combined BR treatments (Pavlović et al., 2024). Similar findings were reported for different microgreens, where LEDs increased TPC by 15-35% compared to darkness, which was attributed to enhanced photosynthetic efficiency and reduced etiolation, which together mitigate oxidative stress and promote the accumulation of secondary metabolites (Zhang et al., 2019; Alrifai et al., 2020; Lee et al., 2023). Notably, the markedly lower TPC in D conditions highlights light’s indispensable role in phenolic induction, leading to compromised antioxidant capacity in etiolated tissues. In comparison with sprouts, microgreens exhibited elevated total plate count (TPC) across various treatments. This pattern reflects photoautotrophic shift, which is known to enhance bioactive profiles. The observed increases in TPC, ranging from 1.5- to 2-fold, are consistent with the findings reported in controlled-environment studies conducted on specialty greens (Martínez-Zamora et al., 2021).

The effect of different LED treatments on TFC in sprouts is of significant interest due to the health promoting properties of flavonoids. LED light quality can significantly influence flavonoid synthesis in sprouts, with blue and white LEDs often showing the most pronounced effects (Nam et al., 2018). In this study, the combined blue-red (BR) treatment demonstrated a significant increase in total flavonoid content (TFC) compared to the other treatments, with an average of 1.046 mg QE/g DW. This value was higher than the red (R) treatment, which had an average of 1.027 mg QE/g DW, and was particularly noteworthy when compared to the blue (B) treatment, which had an average of 0.929 mg QE/g DW. BR treatment also significantly exceeded the levels observed in the D and CW treatments, with average values of 0.952 mg QE/g DW and 0.883 mg QE/g DW, respectively. Red LED light, while generally less effective than blue or white light, still plays a role in flavonoid synthesis. For example, in chickpea and lupin sprouts, different LED lights, including red, influenced isoflavone content, although the relationship between light quality and flavonoid content was not straightforward (Galanty et al., 2022). In common buckwheat sprouts, blue light significantly enhanced the contents of C-glycosylflavones and total flavonoids, as well as antioxidant activities (Nam et al., 2018). The combination of blue and UV-C light improved total flavonoid content in tartary buckwheat sprouts, suggesting that specific combinations of light spectra can be more effective than individual treatments (Ji et al., 2016). Similar to TPC, these results should be interpreted cautiously, as flavonoid synthesis in sprouts is constrained by reserve driven metabolism (Avezum et al., 2023).

The total flavonoid content in pea microgreens demonstrated pronounced spectral-dependent variations, elucidating the nuanced regulatory effects of LED illumination on flavonoid biosynthetic pathways during post-germination development. The maximal TFC observed under combined BR light (1.759 mg QE/g DW), significantly exceeded values under CW (1.307 mg QE/g DW) and D (0.881 mg QE/g DW) conditions while remaining statistically comparable to monochromatic B (1.403 mg QE/g DW) and R (1.406 mg QE/g DW) treatments. Blue light has been found to significantly increase the total flavonoid content in microgreens. For example, in *Ligusticum chuanxiong*, blue light increases the accumulation of flavones and flavonols by upregulating transcription factors such as HY5 and MYBs, crucial for flavonoid biosynthesis (Wu et al., 2023b). In soybean microgreens, blue light, along with UV-A, led to a marked increase in total flavonoid content, with the highest transcript levels of phenolic biosynthesis-related genes observed under these conditions (Zhang et al., 2019). Red light, while less effective than blue light in increasing flavonoid content, still plays a role in the biosynthesis of phenolic compounds. Red light should enhance the growth of microgreens, which can indirectly affect the concentration of flavonoids by altering biomass distribution (Lobiuc et al., 2017).

The HPLC-DAD results clearly show that LED spectral quality has a strong, compound-specific effect on the accumulation of L-ascorbic acid, phenolic compounds and flavonoids in pea sprouts and microgreens, with responses highly dependent on the developmental stage.

L-ascorbic acid was detected at high concentrations, across both growth stages, with significantly higher levels in microgreens than in sprouts. Examining the ascorbic acid content in microgreens, Di Bella et al. (2020) concluded that ascorbic acid levels are higher in the microgreens phase than in earlier and later developmental stages, such as sprouts, baby leaves, and mature plants (Di Bella et al., 2020). This aligns with numerous studies reporting enhanced vitamin C biosynthesis under blue or CW LEDs, due to improved photosynthetic activity (Paglialunga et al., 2024; Rawat et al., 2025). The comparatively lower, yet still substantial, concentrations in sprouts are consistent with their limited photosynthetic capacity and reliance on stored reserves (Ebert, 2022).

Previous studies have demonstrated that monochromatic blue and red lights, compared to white light, significantly affect the synthesis and accumulation of secondary metabolites (Li and Kubota, 2009; Alrifai et al., 2019). The total phenolic content under combined blue-red light declined by 13.3% compared to the mean content observed under blue and red light (Kyriacou et al., 2019a). For some compounds, such as gallic acid or p-coumaric acid, we found higher levels under blue-red light than under red light, which can be explained by the combined signaling of cryptochromes and phototropins (triggered by the blue component) with phytochrome activation (red component) (Trivellini et al., 2023). The phenylpropanoid pathway that produces gallic acid, p-coumaric acid is strongly upregulated by blue lights, through the cryptochrome-mediated transcription factor HY5 that inducing genes such as PAL, C4H, and 4CL (Pech et al., 2022; Palma et al., 2022).

Among phenolic acids, gallic acid was present in all sample sets and exhibited stable concentrations, in microgreens lower than those in sprouts, corroborating previous findings in pea sprouts and other legumes, where gallic acid represents a major phenolic acid regardless of light regime (Borges Martínez et al., 2022). Several studies found a reduction in some total phenolic compounds (catechin and quercetin) of 28% and 64% in soy and peanut seeds, respectively, during germination, probably due to oxidation or enzymatic hydrolysis of these compounds (Cevallos-Casals and Cisneros-Zevallos, 2010). In contrast, p-coumaric acid showed pronounced light-dependent plasticity, particularly in microgreens, where its concentration was strongly enhanced under cool white and blue light but suppressed in darkness. Similar results were observed for cress and mizuna, while a higher concentration under red light was detected in parsley (Kyriacou et al., 2019a).

Flavonoid accumulation further highlighted distinct spectral preferences. In sprouts, (+)-catechin peaked under red light, whereas (–)-epicatechin was maximized under blue light, supporting the concept that different wavelengths selectively regulate branches of flavonoid biosynthesis (Park et al., 2020; Jung et al., 2021). However, the detection of quercetin exclusively under red light in sprouts contrasts with several reports in which blue light enhanced flavanol accumulation, especially in Brassica sprouts and microgreens (Park et al., 2020; Alrifai et al., 2021). This discrepancy underscores both the interspecific and intraspecific specificity of LED responses, particularly in legumes (Vallejo et al., 2003; Kyriacou et al., 2019b). In microgreens, while (–)-epicatechin accumulated abundantly under all light treatments but was absent in darkness, the flavonoid (+)-catechin was detected only under blue light at very high levels, increasing by 285% compared to sprouts. These results are in agreement with the findings of Dueñas et al. (2015), who reported a significant increase in catechin concentration in *P. vulgaris* germinated for 8 days (449.37%). These results align with the consensus that light is essential for flavonoid biosynthesis, with blue wavelengths acting as strong inducers through cryptochrome-mediated signaling (Jung et al., 2021; Menicucci et al., 2025).

Interestingly, quercetin was not detected in sprouts, while its concentrations remained stable across all light treatments in microgreens. This suggests that once photosynthetic tissues are fully functional, quercetin biosynthesis may be less sensitive to spectral variation and more tightly regulated by developmental processes (Kyriacou et al., 2019a). The increase in these compounds during germination could also result from the induction of their synthesis in response to stress (Xu et al., 2018). Vanillic acid was not detected in sprouts, which agrees with another study on peas, where it was found at a low concentration (0.88 µg/g DW) after the ninth day of germination (Borges Martínez et al., 2022). The occurrence of 3, 4-dihydroxybenzoic and vanillic acids predominantly under darkness in microgreens may reflect alternative metabolic routing under carbon limitation or stress conditions, as previously described for etiolated seedlings (Ebert, 2022; Suathong et al., 2024).

Overall, the findings presented in this study corroborate recent evidence from LED-microgreens studies, demonstrating increased vitamin C content and selective phenolic enrichment under blue or combined spectra (Paglialunga et al., 2024; Selelepoo et al., 2025). The results emphasize the necessity of tailoring LED optimization strategies to species, developmental stage, and target phytochemicals, rather than relying on generalized assumptions about spectral effects.

## 5 Conclusions

The present study reveals that the light spectrum and developmental stage significantly affect growth, pigment accumulation and phytochemical parameters in pea sprouts and microgreens. However, these effects varied between developmental stages. In heterotrophic sprouts, responses to light were mainly linked to photomorphogenic signaling rather than fully developed photosynthetic activity, while microgreens, which have functional chloroplasts, showed responses consistent with active photosynthesis. Combined blue-red (BR) and cool white (CW) spectra consistently promoted compact growth, higher dry matter accumulation, and superior overall quality compared to monochromatic or dark conditions. The observed advantages of BR and CW treatments should be interpreted as relative improvements within the specific experimental conditions of this study, rather than as universally optimal lighting strategies, since plant responses to light spectra vary by species, developmental stage, and environmental context. Microgreens outperformed sprouts in all treatments, reflecting their transition to photoautotrophic metabolism and the development of fully functional chloroplasts. BR light was especially effective in enhancing chlorophyll and flavonoid accumulation, highlighting the synergistic effects of red and blue wavelengths. Darkness induced etiolation, resulting in high fresh weight but poor tissue quality and low levels of bioactive compounds. Phenolic and flavonoid accumulation was strongly light-dependent, with CW and BR spectra supporting the highest values in microgreens. Since sprouts are heterotrophic and rely on seed reserve mobilization, while microgreens are photosynthetic and directly responsive to light conditions, optimizing LED spectra is especially critical for maximizing both yield and nutritional quality in microgreens. The implementation of optimized treatments may enhance microgreens quality and production efficiency. This finding is significant because microgreens grown in this way have the potential to be directly utilized for human nutrition purposes. However, these findings relate to compositional changes under controlled conditions and do not directly indicate nutritional bioavailability or dietary impact. Future studies should include a wider range of light intensities and ratios, as well as assessments of nutritional bioavailability, to better define the practical implications of LED lighting in controlled-environment production systems.

## Data Availability

The original contributions presented in the study are included in the article/supplementary material. Further inquiries can be directed to the corresponding author.
